# Transmission of KPC producing *Klebsiella pneumoniae* despite appropriate barrier precautions of an intensive care unit in the Netherlands

**DOI:** 10.1186/1753-6561-5-S6-O25

**Published:** 2011-06-29

**Authors:** B Diederen, C Hattink, M de Groot

**Affiliations:** 1 Regional Laboratory of Public Health Kennemerland, Haarlem, Netherlands; 2 Department of Infection Control, Spaarne Ziekenhuis, Hoofddorp, Netherlands; 3 ICU Department, Spaarne Ziekenhuis, Hoofddorp, Netherlands

## Introduction / objectives

Enterobacteriaceae producing carbapenemases are very rare in the Netherlands and correspond almost exclusively to imported clones from endemic areas. Here, we report an imported case of KPC-carrying *K. pneumoniae* with transmission to another patient.

## Methods

A 68 year old female who was travelling in Greece was admitted to the ICU of the University Hospital of Ioannina in Greece for urosepsis and hypercapnic coma. The patient was transferred to the ICU of the Spaarne Hospital on September 22^nd^ 2010 (day 9). Patient was admitted in strict barrier precautions for MRSA. Intestinal carriage of MDR Enterobacteriaceae was screened at admission and a carbapenem-resistant *K. pneumoniae* strain was isolated from a throat swab. A confirmational PCR was positive for *bla*KPC. On October 14th a routine urine sample from a patient who had been admitted for 15 days at the ICU, tested positive for a genotypically identical KPC producing *K. pneumoniae*.

## Results

Secondary spread was investigated by active surveillance. None of the patients or personnel was found to be positive for KPC-carrying Enterobacteriaceae. No further cases were identified.

## Conclusion

Here we report dissemination of KPC-producing *K. pneumoniae* from a patient repatriated from a Greece ICU despite continuous barrier precautions. We found no evidence for further local spread within our hospital. We stress the importance of early identification and confirmation followed by intensified infection control measures to prevent the dissemination of Enterobacteriaceae with KPC-enzymes.

## Disclosure of interest

None declared.

